# Heterologous microProtein expression identifies LITTLE NINJA, a dominant regulator of jasmonic acid signaling

**DOI:** 10.1073/pnas.2005198117

**Published:** 2020-10-08

**Authors:** Shin-Young Hong, Bin Sun, Daniel Straub, Anko Blaakmeer, Lorenzo Mineri, Jonas Koch, Henrik Brinch-Pedersen, Inger B. Holme, Meike Burow, Hans Jørgen Lyngs Jørgensen, M. Mar Albà, Stephan Wenkel

**Affiliations:** ^a^Department of Plant and Environmental Sciences, University of Copenhagen, 1871 Frederiksberg C, Denmark;; ^b^Copenhagen Plant Science Centre, University of Copenhagen, 1871 Frederiksberg C, Denmark;; ^c^NovoCrops Centre, University of Copenhagen, 1871 Frederiksberg C, Denmark;; ^d^Department of Agricultural and Environmental Sciences, University of Milan, 20133 Milan, Italy;; ^e^Department of Molecular Biology and Genetics, Research Centre Flakkebjerg, Aarhus University, 4200 Slagelse, Denmark;; ^f^DynaMo Centre of Excellence, University of Copenhagen, 1871 Frederiksberg C, Denmark;; ^g^Evolutionary Genomics Group, Research Programme on Biomedical Informatics, Hospital del Mar Research Institute, Universitat Pompeu Fabra, 08003 Barcelona, Spain;; ^h^Catalan Institution for Research and Advanced Studies, 08003 Barcelona, Spain

**Keywords:** microProteins, jasmonic acid, branching

## Abstract

MicroProteins are small, often single-domain proteins that are sequence-related to larger, often multidomain proteins. Here, we used a combination of comparative genomics and heterologous synthetic misexpression to isolate functional cereal microProtein regulators. Our approach identified LITTLE NINJA (LNJ), a microProtein that acts as a modulator of jasmonic acid (JA) signaling. Ectopic expression of *LNJ* in *Arabidopsis* resulted in stunted plants that resembled the decuple *JAZ* (*jazD*) mutant. In fact, comparing the transcriptomes of transgenic *LNJ* overexpressor plants and *jazD* revealed a large overlap of deregulated genes, suggesting that ectopic *LNJ* expression altered JA signaling. Transgenic Brachypodium plants with elevated *LNJ* expression levels showed deregulation of JA signaling as well and displayed reduced growth and enhanced production of side shoots (tiller). This tillering effect was transferable between grass species, and overexpression of *LNJ* in barley and rice caused similar traits. We used a clustered regularly interspaced short palindromic repeats (CRISPR) approach and created a LNJ-like protein in *Arabidopsis* by deleting parts of the coding sentence of the *AFP2* gene that encodes a NINJA-domain protein. These *afp2-crispr* mutants were also stunted in size and resembled *jazD*. Thus, similar genome-engineering approaches can be exploited as a future tool to create LNJ proteins and produce cereals with altered architectures.

## Significance

In plants, hormone signaling is, to some extent, controlled by transcriptional repressors that are degraded in a hormone-dependent manner. These repressor complexes consist of multiple proteins that assemble. MicroProteins are small single-domain proteins that are sequence-related to larger, multidomain proteins and act by disrupting protein complex formation. By forming protein complexes, or by sequestering signaling proteins, microProteins can act as effective modulators of protein activity. Our studies have discovered LITTLE NINJA, a NINJA-related microProtein that modulates JA signaling by attenuating the repression of JA-signaling. Transgenic plants ectopically expressing LITTLE NINJA are shorter and bushier than wild-type plants. Modulating LITTLE NINJA activity may provide strategies for future crop improvement and contribute to finding sustainable solutions for future agricultural production.

To successfully respond to changing environmental conditions, plants have evolved sophisticated signaling systems. These signaling systems consist of proteins that form dynamic higher order structures and rely on protein-protein interactions as well as protein turnover. The formation of protein complexes can be effectively influenced by microProteins. MicroProteins are short single-domain proteins that are sequence-related to larger, often multidomain proteins, and they usually act by engaging the larger related proteins in different protein complexes to control biological processes (1, 2). MicroProtein-coding genes are products of genome evolution events involving either genome duplication or local amplifications followed by evolutionary trimming. Consequently, all microProteins that have been studied to date are sequence-related to the proteins they regulate (2). An example of microProtein function is the modulation of flowering and photomorphogenesis by small B-Box containing miP1a/b-type microProteins that are sequence-related to the CONSTANS (CO) transcription factor (3, 4). MiP1a/b-type microProteins evolved in dicotyledonous plants and besides the B-Box domain, these proteins have an additional TOPLESS-interaction domain. TOPLESS is a transcription corepressor protein that was initially identified to act in auxin signaling (5, 6). It has been shown that the flowering effect strongly depends on the ability of the microProteins to interact with TOPLESS, likely engaging CO in a repressor complex. Interestingly, another microProtein, the MINI ZINC FINGER2 also interacts physically with TOPLESS to control floral meristem termination (7).

TOPLESS does not only act in auxin signaling, but it is also a central component of jasmonic acid (JA) signaling where it engages in a JAZ/NINJA/TPL complex; JA is known as a plant defense hormone. In the absence of JA, the JAZ/NINJA/TPL complex interacts with transcription factors and converts these into repressors of gene expression. When JA is produced or ectopically applied, the CORONATINE INSENSITIVE 1 (COI1) JA-receptor recruits an ubiquitin-SCF-complex to degrade the JAZ-repressor. This degradation alleviates the repressive activity on the transcription factors that can in turn induce JA-responsive gene expression (8). Modulation of JA signaling in different plant species has been associated with altered plant performance such as increased tolerance to salt and drought and higher resistance to pathogens and pests (9). The uncoupling of JA signaling by mutating multiple genes encoding JAZ repressor proteins resulted in slower growing, stunted plants that were less fertile but more resistant to pathogens (10). These and other findings support a model in which JA adjusts defense and growth responses by rewiring the central carbohydrate metabolism.

Here we identified a microProtein with a role in controlling shoot branching. Shoot branching is regulated by different plant hormones and also by microProteins. One of these hormones are brassinosteroids (BRs), which mainly affect plant size through the regulation of cell elongation (11). In rice, BRs also induce bending of the leaf lamina, which can be mimicked by ectopic expression of a helix–loop–helix (HLH)-type microProtein (12, 13). Other plant hormones that have been implicated in controlling the development of side shoots are strigolactones, auxin, and cytokinin. For the latter two, the ratio of cytokinin to auxin has been proposed to be crucial, and recent discoveries support a hierarchical relationship in which cytokinin regulates auxin transport (14). Strigolactones act as suppressors of axillary meristem outgrowth. Hence mutations in *Arabidopsis* genes involved in the production and dissipation of the strigolactone-signal result in plants with a bushy appearance (15–18). Corresponding mutations in orthologous rice genes cause increased numbers of shoots (19, 20), suggesting that strigolactones are ancient branching regulators.

In this study, we used a computational approach to identify microProteins that exist in monocotyledonous plants and validated their biological activity by misexpressing synthetic variants in *Arabidopsis* as a heterologous host. LITTLE NINJA (LNJ) a microProtein related to NINJA proteins caused when overexpressed in *Arabidopsis*, Brachypodium, barley, and rice a reduction of plant size. Additionally, transgenic plants also appeared bushier. The sequence relationship to NINJA prompted us to investigate a possible role in JA signaling; in agreement, we found that LNJ can modulate JA-signaling. The latter effect is likely through the poisoning of the pool of NINJA proteins required for the repression of JA-signaling. Hormone profiling revealed substantial changes in transgenic plants that likely account for the bushy appearance. Our finding that the LITTLE NINJA effect is transferrable between distant cereal crops makes it a promising breeding target. Thus, our results have significant implications for the complex regulation of JA-mediated transcriptional regulation and its potential role in future crop improvement.

## Results

### Identification of LNJ, a Protein Isoform Related to NINJA.

**Fig. 1.**
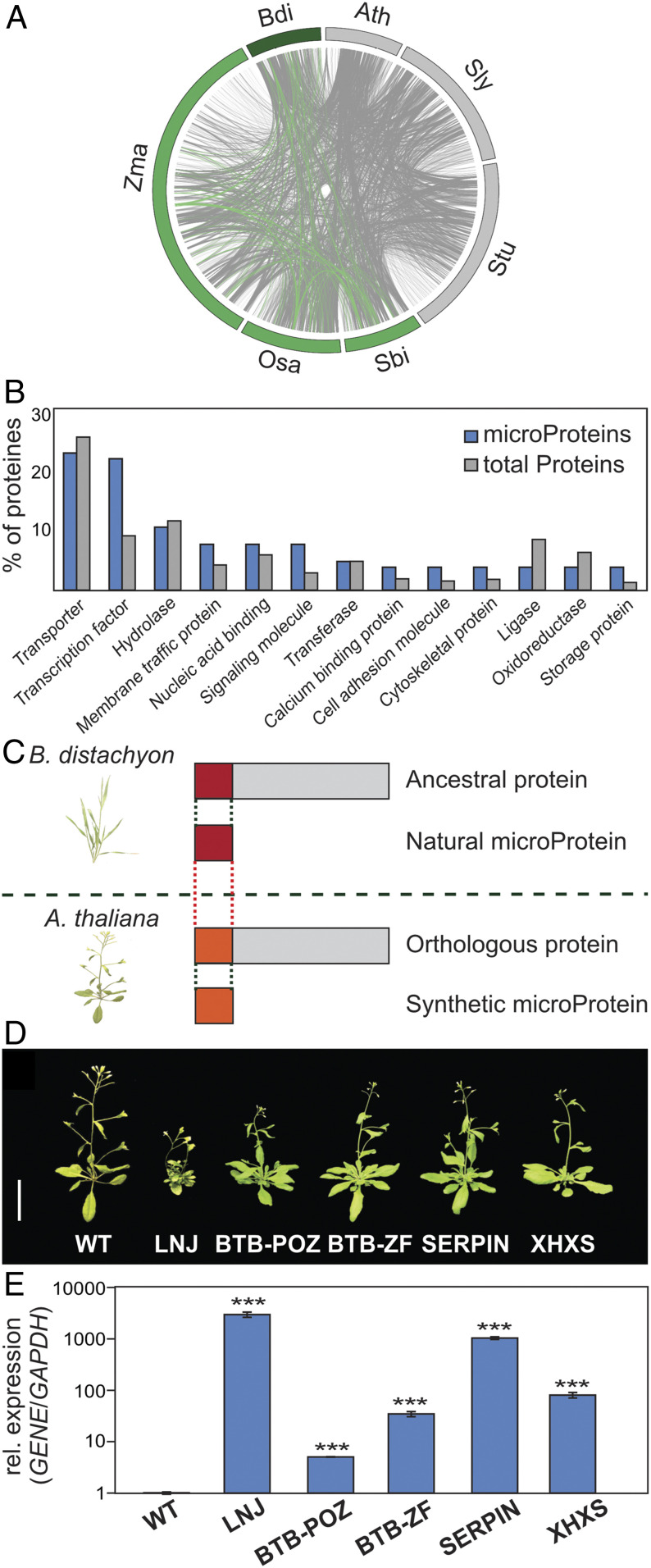
Identification of monocot-specific microProtein candidates and production of synthetic *Arabidopsis* microProteins. (*A*) Circos plot depicting evolutionary relationships of plant microProteins. Connections show conservation between species based on OrthoFinder. Dark-green arc indicates *B. distachyon21-3* (Bdi). Green arcs indicate representative monocotyledonous plants; *Zea mays* (Zma), *O. sativa* (Osa), and *Sorghum bicolor* (Sbi). Gray arcs indicate representative dicotyledonous plants; *Arabidopsis thaliana* (Ath), *Solanum lycopersicum* (Sly), and *Solanum tuberosum* (Stu). Green lines specify the fraction of monocot-specific microProteins while gray lines specify microProteins present in all plant genomes. (*B*) Functional annotation of microProteins by protein classification using PANTHER (www.pantherdb.org/). The *y* axis indicates the percentage of unigenes in specific functional clusters. (*C*) Natural microProteins investigated here are monocot specific and possess conserved ancestral proteins in the *Brachypodium* genome. The *Arabidopsis* genome encode monocot-specific microProtein ancestors (orthologous protein). Based on the structure of the natural Brachypodium microProtein, *Arabidopsis* synthetic microProteins were designed. (*D* and *E*) Phenotype (*D*) and relative expression levels (*E*) of the transgenic plants overexpressing synthetic microProtein genes. *Arabidopsis glyceraldehyde 3-phosphate dehydrogenase (GAPDH*) expression was used as reference gene for qRT-PCR and values were calibrated to the wild-type control (expression set to 1). Values are the means ± SD *P < 0.05, **P < 0.005, ***P < 0.0005 determined by Student’s *t* test. *n* = 3. (Scale bar, 10 cm.)

To identify new regulators of development, we employed a computational approach to detect monocot microProteins. Using the MiPFinder pipeline (21) we developed earlier, we first identified plant-specific microProteins and focused on the fraction that is present in monocotyledonous plants (Fig. 1*A*). Here, we focused on microProteins that can be identified in one of the monocotyledonous reference genomes (rice, sorghum, maize, or Brachypodium), but not in any of the dicotyledonous reference genomes (*Arabidopsis*, potato, tomato). In-depth analysis of this subset of microProteins encoded in the *Brachypodium distachion* genome revealed an enrichment for microProteins related to transcription factors and signaling molecules (Fig. 1*B*). We then decided to use transformation-efficient *Arabidopsis* plants to prescreen regulators of interest. In the Brachypodium genome, we found genes encoding microProteins as well as the corresponding ancestral, larger proteins. For the selected monocot-specific microProteins, we only found the ancestral larger proteins in *Arabidopsis* but not in the corresponding microProteins (Fig. 1*C*). This enabled us to develop so-called synthetic microProteins predicted to interfere with the ancestral proteins in *Arabidopsis*, potentially causing deviations from the normal growth behavior. In an initial screening, we overexpressed five such synthetic microProtein candidates in transgenic *Arabidopsis* plants. This strategy resulted in the identification of one candidate, which we named *LNJ*. Sequence analysis of *LNJ* revealed that it is closely related to the *Arabidopsis ABI5-BINDING PROTEIN2* (*AFP2*) gene that encodes a protein containing a NINJA domain. Overexpression of full-length *AFP2* has recently been shown to delay flowering while the *afp2* mutant plants flowered earlier than wild-type (22). These phenotypes contrast what we observed in transgenic *LNJ-OX* plants that were stunted and lost apical dominance (Fig. 1*D*). This indicates that the dominant-negative function of LNJ differs from the biological functions of AFP2.

A further inspection of the LNJ origin uncovered that it is as expected not present in *Arabidopsis*, tomato, and potato, but it is also not monocot specific. Furthermore, LNJ is not encoded by an individual gene but is a potential splice isoform of a larger NINJA protein that had not been correctly annotated in the Brachypodium genome (*SI Appendix*, Fig. S1). Similar to Brachypodium, the same *LNJ* isoform is also annotated in maize, indicating that LNJ-like microProteins might be produced by alternative splicing. Inspection of the LNJ protein sequence in dicot genomes revealed that some species encode small LNJ-like microProteins as single copy genes. However, their evolutionary origin cannot be easily traced back and it seems that these microProteins evolved independently from different types of NINJA protein precursors.

The misexpression of other synthetic microProtein candidates produced phenotypically normal plants under standard growth conditions. All transgenic plants had high levels of synthetic microProtein messenger RNAs (mRNAs), supporting that they all were genuine overexpressors (Fig. 1*E*). Our strategy of using *Arabidopsis* as a heterologous host for ectopic synthetic microProtein expression proved to be successful and it can be used for future microProtein characterization of species where transformation is not easy or not yet possible.

### *Arabidopsis LNJ-OX* Plants Resemble *jaz-D* Mutant Plants.

**Fig. 2.**
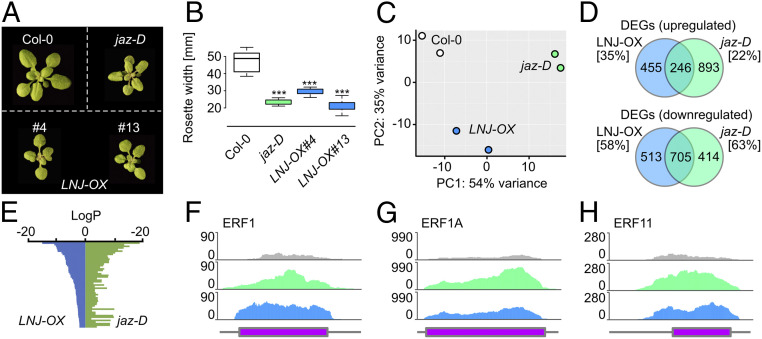
Ectopic expression of the *Arabidopsis* LNJ protein changes plant size and transgenic plants resemble *jaz-D* mutant plants. (*A*) Picture of representative 20-d-old seedlings. (*B*) Rosette width of wild-type Col-0, *jaz-D*, and two *LNJ-OX* transgenic plants. Box plots show the observed experimental data. Rosette width was determined after the transition to flowering. ***P < 0.0005 determined by Student’s *t* test. *n* = 11 to 13. (*C*) Principle component analysis (PCA) of the gene expression (regularized logarithm transformed count data) between the different RNA-seq libraries. Plotted is the percentage of variance for each component for each of the two biological samples per genotype. (*D*) Venn diagram showing the overlap of differentially expressed genes (log2FC > +1 and < −1, BH-adj. *P* value < 0.01) compared to the Col-0 wild-type. Numbers in brackets depict the overlap between the two datasets. (*E*) Comparative gene ontology analysis of genes down-regulated in *LNJ-OX*, and *jaz-D* shows a large overlap in genes and GO categories enriched in the two genetic backgrounds. (*F*–*H*) RNA-seq read coverages of representative candidate genes showing expression levels in the three different backgrounds.

Having established that overexpression of *LNJ* in *Arabidopsis* causes a stunted growth phenotype resembling the recently described *jazD* decuple mutant (10), we assessed growth of the latter mutant in comparison to Col-0 wild-type and *LNJ-OX* plants. In agreement with previous findings (10), we observed a stunted growth phenotype of *jazD* bearing resemblance to *LNJ-OX* plants (Fig. 2 *A* and *B*). Multiple pathways have been described that can affect plant size. We therefore isolated RNA from Col-0 wild-type, *jazD,* and *LNJ-OX* plants to compare their individual transcriptomes (Dataset S1). Principle component analysis of the data revealed that individual replicates grouped together, indicating reproducible but distinct datasets (Fig. 2*C*). The analysis of differentially expressed genes (DEGs) identified 701 genes that were ectopically up-regulated in *LNJ-OX* plants and 1,139 genes that were highly expressed in *jazD* compared to Col-0 wild-type (Fig. 2*D*). In total, 246 genes were up-regulated in both *LNJ-OX* and *jazD*, corresponding to 35% and 22%, respectively. The analysis of down-regulated transcripts discovered 1,218 DEGs in *LNJ-OX*, 1,119 DEGs in *jazD*, and an overlap of 705 genes. The latter translates into an overlap of 58% in *LNJ-OX* and 63% in *jazD* (Fig. 2*D*). A comparative analysis of the down-regulated genes revealed also a significant overlap with regards to deregulated gene ontology categories (Fig. 2*E*). These results indicate that the stunted growth phenotype could be due to the deregulation of similar genes and is likely a result of ectopic JA signaling. Previous RNA sequencing (RNA-seq) studies of *jazD* have identified a number of *ETHYLENE RESPONSE FACTOR* (*ERF*) genes that were highly expressed in *jazD* (10). We checked the expression of three *ERFs* (*ERF1*, *ERF1A*, and *ERF11*) and found these to be strongly induced in both *jazD* and *LNJ-OX* plants (Fig. 2 *F*–*H*), further supporting the idea that these plants are affected in the same signaling pathway.

### LITTLE NINJA Is Functional in Brachypodium.

**Fig. 3.**
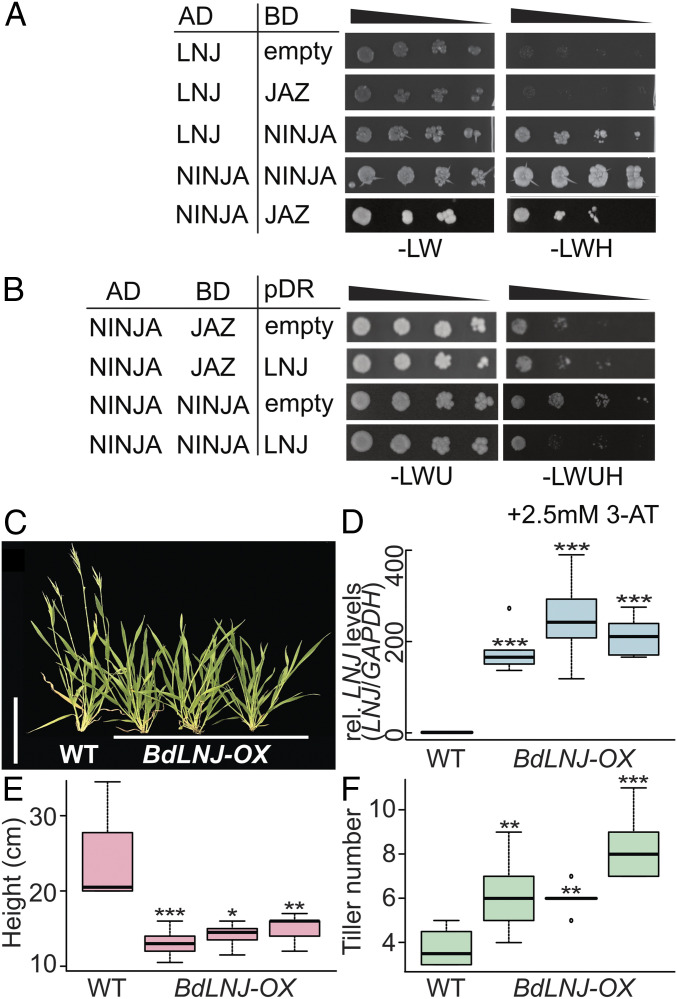
LNJ interacts with NINJA and ectopic expression alters the shoot architecture of *Brachypodium* plants. (*A*) Yeast-two-hybrid interaction of LNJ (BRADI1g69970) fused to the Gal4 AD and the Gal4 BD and fusions of BD to JAZ (BRADI1g21490) and NINJA (BRADI2g11790). LNJ interacts with NINJA as indicated by growth on selective medium (−LWH). No interaction was observed between LNJ and JAZ but NINJA and JAZ interact and NINIJA can also homodimerize as indicated by growth on selective medium of yeast expressing both AD-NINJA and BD-NINJA or BD-JAZ. (*B*) Yeast-three-hybrid interaction assay. Yeast expressing AD fusions to NINJA and BD fusions to JAZ or NINJA were transformed with either pDR-empty or pDR-LNJ. NINJA/JAZ and NINJA/NINJA dimerization was observed on quadruple dropout medium in the presence of the empty vector, in the presence of LNJ; NINJA/JAZ heterodimerization remained unaffected but homodimerization of NINJA was weakened as indicated by a reduced ability to grow on the selective medium. (*C*) Picture of 4-wk-old plants of the wild-type Bd21-3 and three independent *BdLNJ-OX* lines grown under long day (LD) photoperiod (22/18 °C, 16 h light/8 h dark). (Scale bar, 10 cm.) (*D*) Relative expression levels of *BdLNJ* mRNA in wild-type and independent T3 transgenic plants determined by qRT-PCR. Expression levels, determined as relative quantities and normalized against *Brachypodium GAPDH* gene, are depicted as box plots, *n* = 3. (*E*) Quantification of plant height depicted as box plots in wild-type and three independent *BdLNJ-OX* lines grown under LD photoperiod. (*F*) Quantification of tiller number depicted as box plots in wild-type and three independent *BdLNJ-OX* lines grown under LD photoperiod. *P < 0.05, **P < 0.005, ***P < 0.0005 determined by Student’s *t* test. *n* = 15 to 20.

We investigated the sequence of LNJ and the *Bd*LNJ microProtein in more detail and found that it contains the “Domain C” of NINJA proteins (*SI Appendix*, Fig. S2). NINJA proteins have known roles in jasmonic acid (JA) signal transduction and Domain C allows NINJA to interact with JAZ proteins (8). Similar to the situation in auxin signaling, JAZ/NINJA proteins recruit the TOPLESS (TPL) corepressor to transcription factors, here MYC-type factors that regulate JA response genes. Thus, downstream target genes are constantly repressed in the absence of JA. Once JA is present, it initiates the degradation of JAZ through the proteasome pathway, which results in the induction of JA-responsive genes (23, 24). Based on the protein sequence of LNJ, we hypothesized that it would influence jasmonic acid signaling either by shielding JA-responsive transcription factors from being repressed by the NINJA/TPL complex or by sequestering JAZ/NINJA/TPL directly (*SI Appendix*, Fig. S3). We tested in yeast whether LNJ interacted with either JAZ or NINJA and found an interaction with NINJA but not with JAZ (Fig. 3*A*). We also detected that NINJA can dimerize in yeast and LNJ can influence the dimeric state of NINJA repressors but not the heteromeric state of NINJA/JAZ (Fig. 3*B*). A negative influence on the formation of NINJA complexes could result in ectopic JA signaling activity in the absence of the hormone. Jasmonic acid has diverse roles in regulating developmental processes such as seed germination, root development, and senescence, but it is best known as a plant defense hormone (25).

To determine whether ectopic expression of the Brachypodium *LNJ* (*BdLNJ*) would also affect growth and development of Brachypodium plants, we generated transgenic lines overexpressing the LNJ microProtein (*BdLNJ-OX*). Phenotypic analysis of transgenic T2 and T3 plants revealed a significant reduction of the height of adult plants that coincided with increased levels of transgene expression (Fig. 3 *C*–*E*). Besides having a small stature, *BdLNJ-OX* transgenic plants developed more axillary shoots compared to nontransgenic wild-type plants (Fig. 3*F*). Taken together, BdLNJ acts as a dominant master regulator of plant stature and leads to shorter, more branched Brachypodium plants.

To test if JA influences the size and branching of Brachypodium, we treated wild-type and *BdLNJ-OX* plants with 100 μM methyl jasmonate (MeJA) by spraying. Notably, the size of wild-type plants was strongly affected by the MeJA treatment and treated plants resembled *BdLNJ-OX* plants. On the contrary, *BdLNJ-OX* plants showed no further reduction in growth and thus appeared unresponsive to MeJA (*SI Appendix*, Fig. S4 *A* and *B*). We next analyzed whether the degrees of shoot branching of wild-type and *BdLNJ-OX* plants treated with either MeJA or a mock solution was affected. No increased numbers of axillary branches were observed in response to MeJA treatment (*SI Appendix*, Fig. S4*C*), indicative of additional or parallel functions of BdLNJ. We conclude that JA had an impact on plant size and the finding that *BdLNJ-OX* plants were not responsive to ectopic MeJA application supports the idea that JA signaling is affected in *BdLNJ-OX* transgenic plants, contributing to the phenotypic changes we observed.

### LITTLE NINJA Affects JA Signaling in Brachypodium.

**Fig. 4.**
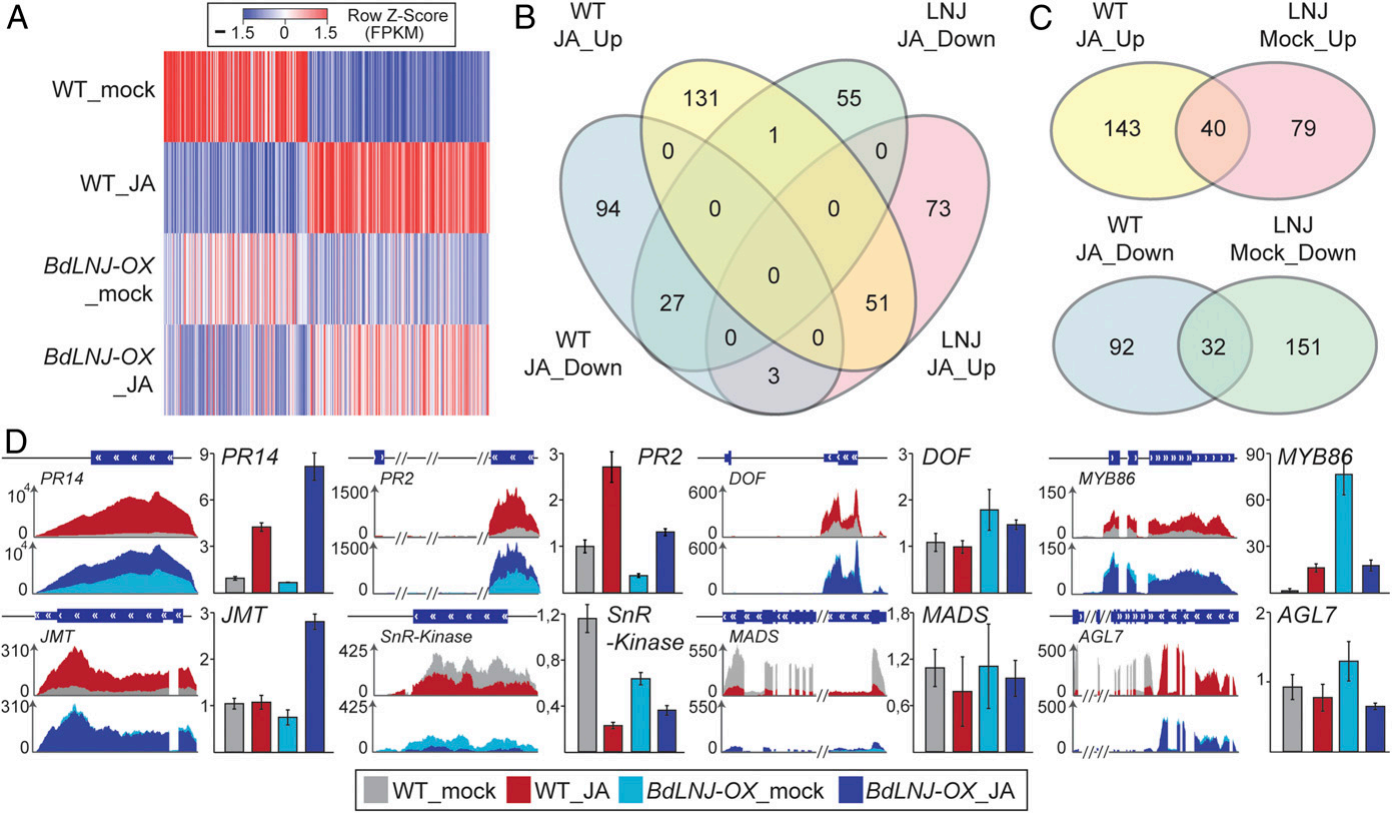
BdLNJ globally affects jasmonic acid signaling. (*A*) Heat map of differentially expressed genes in wild-type *Brachypodium* (WT_mock/WT_JA) and *BdLNJ-OX* plant (*LNJ-OX*_mock/*LNJ-OX*_JA) with and without MeJA (mock/JA). The color scale indicates gene expression level (FPKM values). For each genotype and treatment, two biological samples were analyzed. (*B*) Venn diagram showing DEGs expressed in the four samples. WT indicates wild-type *Brachypodium* and *LNJ* indicates *BdLNJ-OX* plant. The number of genes of JA_UP or JA_Down indicates up-regulated or down-regulated genes in response to MeJA treatment. (*C*) Venn diagrams showing genes that were significantly induced after MeJA treatment (FDR ≤ 0.05). Induced genes were defined as transcripts with fold changes ≥2 based on gene expression levels between WT JA_UP and *LNJ* Mock_UP, between WT JA_Down and *LNJ* Mock_Down, respectively. (*D*) Validation of candidate genes identified by RNA-Seq using qRT-PCR. RNA-Seq read coverages of genes that are up- or down-regulated in response to MeJA treatment. Gene models depict exons in blue. QRT-PCR analysis of differential gene expression in MeJA treated wild-type *Brachypodium* and *BdLNJ-OX* plant, *n* = 3. RNA-seq read maps and qRT-PCRs show mock-treated wild-type *Brachypodium* in gray and wild-type MeJA-treated samples in red. The mock-treated *BdLNJ-OX* samples are shown in light blue and corresponding MeJA-treated samples in dark blue.

We had implicated BdLNJ in jasmonic acid signaling and therefore sought to determine whether transgenic *BdLNJ-OX* plants have an altered molecular response to JA. To test this, we performed a mRNA-sequencing experiment with RNA isolated from wild-type and transgenic *BdLNJ-OX* plants that were either treated with MeJA or a mock solution. Our analysis discovered 183 genes to be significantly up-regulated by MeJA treatment in wild-type plants and 127 genes to be up-regulated in transgenic *BdLNJ-OX* plants (Fig. 4 *A* and *B* and Dataset S2). On the contrary, we found 124 genes in wild-type and 183 genes in *BdLNJ-OX* plants to be down-regulated in response to MeJA (Fig. 4 *A* and *B*). The global analysis of the MeJA-influenced transcriptome in *BdLNJ-OX* plants compared to wild-type showed significant changes (Fig. 4 *A* and *B*). Interestingly, in contrast to *Arabidopsis*, we observed no significant transcriptional changes of *JAZ* genes in response to MeJA treatments. On the other hand, in-depth analysis of the 183 genes up-regulated by MeJA in wild-type revealed that as many as 40 genes (corresponding to around 22%) are ectopically induced in *BdLNJ-OX* plants (Fig. 4*C*). Similarly, for the 124 genes down-regulated by MeJA treatment of wild-type plants, we find 32 genes (corresponding to 26%) to be expressed at reduced levels in mock-treated *BdLNJ-OX* plants. Genes regulated by MeJa or by ectopic *BdLNJ* expression encode enzymes such as JASMONIC ACID METHYLTRANSFERASE (JMT), diverse transcription factors, such as the Brachypodium ortholog to MYB86, and enzymes such as β-1,3-GLUCANASE (Dataset S2). Some of these factors, such as JMT and β-1,3-GLUCANASE have established roles in jasmonic acid signaling in *Arabidopsis* (26, 27). We investigated gene expression changes that were initially observed by mRNA-seq by using qRT-PCR (Fig. 4*D*). We found that some genes change as detected by mRNA-seq, others such as *JMT* and the *MADS* genes did not show the expected changes, which could be a result of PCR bias. We conclude that LNJ affects jasmonic acid signaling, likely by interfering with the JAZ/NINJA/TPL repressor complex causing JA-response genes to be expressed as if plants were treated with MeJA.

To investigate if the changes we observed by mRNA-seq correlate with metabolic alterations, we measured the abundance of the major plant hormones by mass spectrometry. The altered JA transcriptional network in plants ectopically expressing *BdLNJ* correlated in all three transgenic lines with significantly decreased levels of the active signaling compound JA-isoleucine. In addition, we observed overall perturbations in the hormone profiles of *BdLNJ-OX* plants, including decreased levels of cytokinins that could impact the indole-3-acetic acid (IAA):cytokinin ratio (*SI Appendix*, Fig. S5). A skewed IAA:cytokinin ratio might be causal for observed changes in shoot branching. In addition to the changes in hormone levels, we also observed a repression of genes in JA- and SA-related responses in transgenic *BdLNJ-OX* plants (*SI Appendix*, Fig. S6), which further supports a role for LNJ in hormone signaling. Taken together, BdLNJ acts as a modulator of JA-responsive gene regulation, accounts for around 25% of all JA-induced gene expression changes, and ectopic expression of LNJ affects plant hormone levels, indicating that LNJ is a regulator of JA signaling.

### The LITTLE NINJA Effect Is Transferrable to Cereal Crops.

**Fig. 5.**
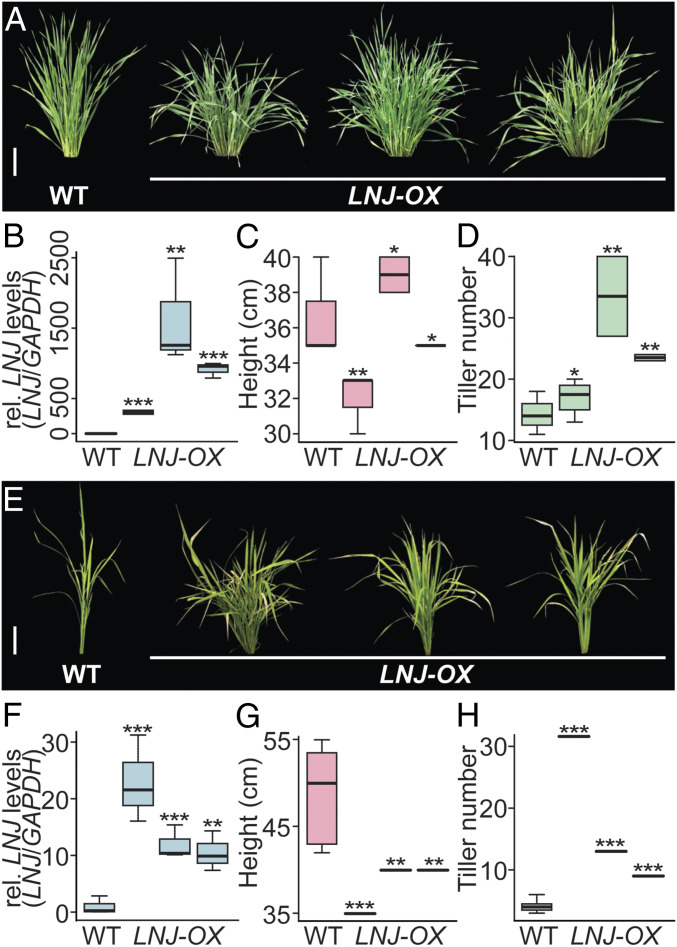
LNJ influences shoot architecture of crop plants. (*A*) Picture of 2-mo-old barley plants. Wild-type barley (*H. vulgare* cv. Golden Promise) and three independent *LNJ-OX* lines grown under LD photoperiod (20/15 °C, 16 h light/8 h dark). (Scale bar, 10 cm.) (*B*) Relative expression levels of *LNJ* mRNA in wild-type and independent T2 transgenic plants which were determined by qRT-PCR. Expression levels, normalized against barley *GAPDH* gene, are depicted as box plots; *n* = 3. (*C* and *D*) Quantification of height (*C*) and tiller number (*D*) are depicted as box plots in wild-type and three independent *LNJ-OX* lines grown under LD photoperiod. *n* = 8 to 15. (*E*) Picture of 3-mo-old representative rice plants. Wild-type rice (*Oryza sativa* cv. Nipponbare) and three independent *LNJ-OX* lines grown under SD photoperiod (28/25 °C, 12 h light/12 h dark). (Scale bar, 10 cm.) (*F*) Relative expression levels of *LNJ* mRNA in wild-type and independent T0 transgenic plants which were determined by qRT-PCR. Expression levels, determined as relative quantities and normalized against rice *ACTIN* gene, are depicted as box plots; *n* = 3. (*G* and *H*) Quantification of height (*G*) and tiller number (*H*) are depicted as box plots in wild-type and three independent *LNJ-OX* lines grown under SD photoperiod. *P < 0.05, **P < 0.005, ***P < 0.0005 determined by Student’s *t* test.

To understand whether BdLNJ acts as a universal regulator of plant architecture, we also generated transgenic barley and rice plants overexpressing *BdLNJ*. All transgenic barley plants that we generated overexpressed the transgene, but in comparison to Brachypodium were not consistently stunted (Fig. 5 *A*–*C*). We used the cultivar *Golden Promise* for transformation, which in comparison to Brachypodium is a domesticated crop plant and already shorter than wild, nondomesticated relatives. Golden Promise carries a loss-of-function mutation in HvDep1, an AGG3-type subunit encoding gene that positively regulates culm elongation and seed size in barley (28). When assessing the tiller numbers, we do however find significant increases in the number of axillary shoots and transgenic plants had a much bushier appearance. Thus, BdLNJ is functional in barley. Brachypodium and barley are, together with wheat, oat, and ryegrass, part of the *Pooideae* subfamily. To determine whether similar effects could also be achieved in a more distantly related crop plant, we generated transgenic rice (*Oryzoideae*) overexpressing *BdLNJ*. All transgenic plants that were recovered after transformation overexpressed the transgene and produced rice plants that were significantly stunted with increased numbers of tillers (Fig. 5 *E*–*H*). Taken together, these results indicate that even the growth behavior of domesticated germplasm can be further optimized by ectopic LNJ expression.

Disturbances of hormone pathways, especially jasmonic acid could potentially lead to an increased pathogen susceptibility. Therefore, we tested if transgenic *LNJ-OX* plants respond differently to pathogen attack. Inoculation with the barley pathogen *Pyrenophora teres* revealed no difference between wild-type barley plants and the transgenic *LNJ-OX* plants (*SI Appendix*, Fig. S7). Thus, it appears that plants with constitutive *LNJ* levels produce more tillers and are not compromised in their response to pathogen attack.

### Creating a LITTLE NINJA Gene in *Arabidopsis*.

**Fig. 6.**
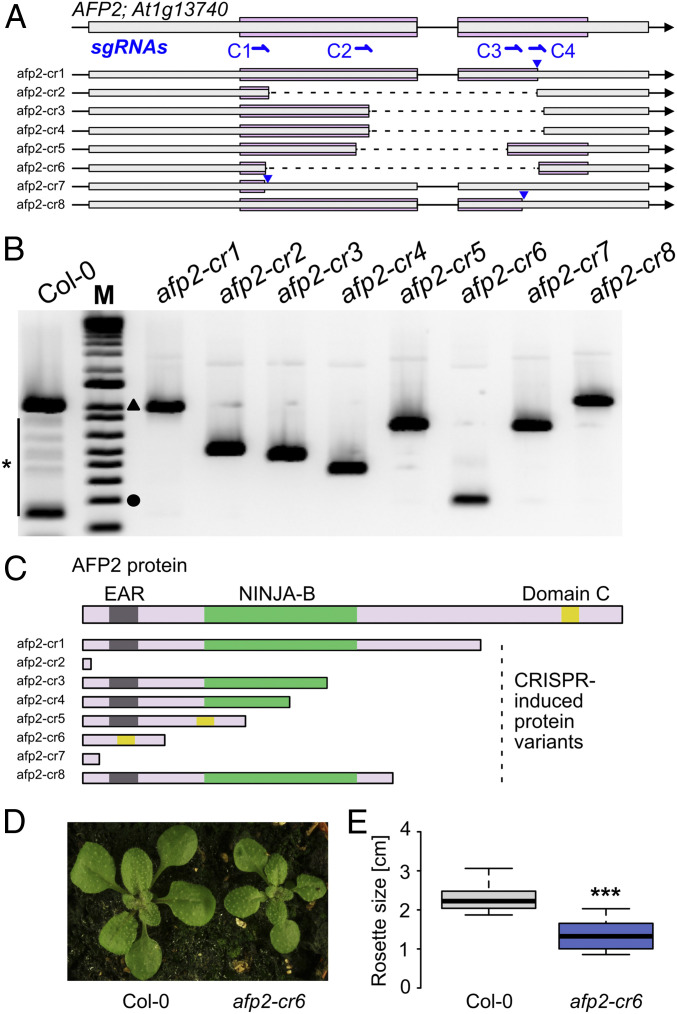
Creation of a LITTLE NINJA microProtein in *Arabidopsis*. (*A*) Gene model depicting the *AFP2* locus with the coding sequence highlighted in pink. Four sgRNAs (C1 to C4) were designed and transformed as pools into *Arabidopsis* plants. Eight CRISPR-induced mutants (*afp2-cr1-cr8*) were identified. Dashed lines indicate deleted regions. Triangles depict point mutations. (*B*) RT-PCR showing amplification of the *AFP2*-coding region in wild-type and the eight *afp2*-CRISPR mutant plants. Star indicates spurious PCR products in wild-type. The circle depicts the 200 bp marker and the black triangle the 1.0 kb marker. (*C*) Protein model depicting the full-length AFP2 protein and the protein domains. The eight CRISPR mutants give rise to different protein isoforms. (*D*) Growth analysis of wild-type and *afp2-cr6* T2 mutant plants revealed that *afp2-cr6* plants showed reduced rosette sizes. (*E*) Rosette width of wild-type Col-0 and *afp2-cr6* of 3-wk-old plants. Box plots show the observed experimental data. *n* = 8, ***P < 0.0005 determined by Student’s *t* test.

Overexpression of a LITTLE NINJA isoform of *AFP2* caused a significant reduction of the rosette size in transgenic *Arabidopsis* plants (Fig. 2 *A* and *B*). This phenotype is different from the *afp2* mutant or the overexpression of the full-length protein that has been shown to display alterations in flowering time (22). To test if a functional LITTLE NINJA protein could be engineered into the *Arabidopsis* genome, we employed a clustered regularly interspaced short palindromic repeats (CRISPR)/Cas-9 approach and designed single guide RNAs that would recognize regions after the translation start site and regions close to the Domain C (Fig. 6*A*) potentially cleaving out the EAR and NINJA domains. After transformation of the pooled single guide RNAs (sgRNAs), we obtained transgenic plants in which we detected chromosomal deletions in the *AFP2* gene (Fig. 6*B*). Sequencing of the RNA isoforms revealed several AFP2 protein isoforms with altered domain composition (Fig. 6*C*) including one isoform (*afp2-cr6*) in which the reading frame was maintained but the entire amino-terminal region including the EAR and NINJA domains was deleted. Inspection of the *afp2-cr6* T2 deletion mutants revealed a significant reduction in the rosette size of mutant compared to wild-type plants (Fig. 6 *D* and *E*), and *afp2-cr6* mutants resembled *jazD* mutant plants. Together, these findings show that microProteins can be engineered from individual genes to establish regulatory feedback loops.

## Discussion

Here we report the identification of the LNJ microProtein involved in the modulation of jasmonic acid signaling. We initially identified LNJ as a potentially monocot-specific microProtein, which means that we did not identify LNJ or LNJ-related proteins in our reference dicot genomes (here *Arabidopsis*, tomato, and potato). Closer analysis of the LNJ protein sequence using Basic Local Alignment Search Tool (BLAST) searches revealed, however, that LNJ-like proteins can be identified in other dicot genomes and it is thus not monocot specific. We initially identified LNJ because of a misannotation of a gene encoding a larger NINJA-domain protein where the first exon was excluded (*SI Appendix*, Fig. S1). To study the loss of *LNJ* function in Brachypodium, we also attempted to generate respective mutants but it is not yet possible to target a specific splice isoform because both isoforms are identical except for their length. Introduction of CRISPR guide RNAs related to *LNJ*, which will affect both the long and the short isoforms, resulted in the isolation of only few transgenic calli that after shoot-induction failed to induce roots and perished during the regeneration process (*SI Appendix*, Fig. S8). Thus, it is currently unclear whether an LNJ-type protein is formed in Brachypodium and what its function would be. The prediction that LNJ-type microProteins may exist in different plant genomes as individual genes suggests that the mechanism of modulating or buffering JA signaling might be evolutionary conserved.

We provide evidence that the Brachypodium LNJ protein acts as a dominant regulator of plant stature. Ectopic LNJ expression alters JA signaling, which is likely partially responsible for the growth phenotypes that we have observed. This is in agreement with a recent metabolomic analysis of a barley brassinosteroid-insensitive mutant that showed a semidwarf phenotype and had altered levels of JA and gibberellic acid (29). The dwarf phenotypes we observed could therefore at least be partially due to disturbances in the hormone homeostasis. Our hormone profiling comparing Brachypodium wild-type and *LNJ-OX* transgenic plants demonstrated that several hormones are significantly different. The level of JA-Ile (isoleucine), the active signaling compound of jasmonic acid, was reduced in *LNJ-OX* plants compared to wild-type (*SI Appendix*, Fig. S5). This negative correlation could be a consequence of inducing JA-signaling in the absence of the hormone and a concomitant negative feedback that curbs JA biosynthesis. In addition, it is well established that the ratio of auxin to cytokinin has a profound impact on plant development and morphogenesis. The observation that the levels of both auxin and different cytokinins were considerably altered (*SI Appendix*, Fig. S5) suggests that the branching phenotype might be a consequence of the simultaneous alteration of different hormone levels. The analysis of RNA-sequencing data (Dataset S2), however, does not contain obvious candidates that were induced in *BdLNJ-OX* plants and involved in specific hormonal signaling pathways. Interestingly, in comparison to *Arabidopsis* (8, 30), we did not detect changes in *JAZ* expression in response to MeJA treatment in Brachypodium. This could either indicate that we missed the timepoint of *JAZ* induction in our experiments or that JA-signaling is different in grasses.

The finding that the function of LNJ is transferrable between crop plants (here, rice and barley) indicates that LNJ can function as a general growth regulator, at least in the BEP (Bambusoideae-Ehrhartoideae-Pooideae) clade. Interestingly, overexpression of *LNJ* did not result in an increased pathogen resistance, a trait that had been observed in higher order *jaz* mutants (10).

Recently, a promoter editing approach using CRISPR/Cas9 has been used to increase phenotypic diversity, resulting in edited plants with altered expression levels of candidate genes (31). As we have shown in *Arabidopsis*, LITTLE NINJA-like proteins can be created by deleting parts of the coding sequence (Fig. 6 *A*–*D*). Similar genome-engineering approaches in crop plants can be used to create one or more LITTLE NINJA proteins. Additional promoter-hacking approaches could then lead to crop plants with enhanced levels of *LNJ* expression that will likely correlate with altered architectural traits.

The comparison of *LNJ-OX* and *jazD* mutant plants revealed that both plants show reduced growth (Fig. 2). However, *jazD* mutants are also less fertile than wild-type plants and more resistant to pathogens, and these traits have not been observed in *LNJ-OX* plants. Thus, it seems that despite the phenotypic similarities, differences exist that could result from interferences with different signaling pathways. Molecularly, we detected a significant overlap of commonly deregulated genes, which suggests that LNJ could be used as a tool to affect JA signaling in a cell type-specific fashion. Since it is challenging to remove 10 *JAZ* genes in a given cell type, but it is feasible to misexpress one LNJ, tissue- or cell-specific JA responses could be dissected using LNJ-like isoforms.

Finally, because of its functional conservation, LITTLE NINJA-like proteins can be engineered and could be used as a universal tool to control architectural traits of many major crops including wheat, maize, sorghum, and sugar cane.

## Materials and Methods

### Plant Material, Growth Conditions, Measurements of Agronomic Traits.

Seeds of the *Arabidopsis* Col-0 ecotype were stratified at 4 °C for 3 d and transplanted into soil and grown at 22 °C in a growth chamber with a 16 h/8 h (day/night) photoperiod and ∼ 65% relative humidity (RH).

*B. distachyon* inbred line *Bd21-3* was stratified at 4 °C for 5 d and transplanted into soil and grown at 24/16 °C in a growth chamber with a 16 h/8 h (day/night) photoperiod and ∼ 60% RH. Plant height was determined by measuring stem lengths of plants 4 wk after germination.

The barley cultivar used in this study is Golden Promise (*Hordeum vulgare*). Barley seeds from wild-type plants and the transgenic plants imbibed in darkness for 3 d at 4 °C and transplanted into soil. Plants were grown at 20 °C and a 16 h photoperiod (400 to 500 μE), followed by 8 h at 15 °C without light and a relative humidity of 65%.

The rice cultivar used in the experiments is Nipponbare (*Oryza sativa*). Rice seeds from the control plants and the transgenic plants were imbibed in darkness for 3 d at 4 °C and then were grown at 28 °C, and a 12 h photoperiod (350 μE), followed by 12 h at 25 °C without light and a RH (80% for day, 75% for night). For disease-resistant assays, barley plants were treated with *P. teres* as described earlier (32).

*Arabidopsis* transgenic plants (T3) were grown at 22 °C. Phenotype analysis was performed with eight individual plants per line. *Brachypodium* plants of 4-wk-old plants (T3) were grown at 24/16 °C. Height and tiller number were analyzed with 15 to 20 individual plants per line. Total spikelet numbers were determined when the seeds were harvested. The 2-mo-old barley plants (T2) with individual plants per line (8–15) were measured for height and tiller number. The height and tiller number were determined for T0 transgenic rice plants.

### Identification and Classification of MicroProtein Candidates.

Identification of *B. distachyon* microProtein candidates was done with MiPFinder v1.1 (21) using *B. distachyon* v3.1 genome (33), and conservation in other genomes was determined by OrthoFinder v0.3.0 (34) using published microProtein lists (21). Only microProtein candidates containing interaction domains according to Pfam v28 (pfam.xfam.org/) and iPfam v1.0 (35) were retained. Evolutionary conservation of microProtein candidates was visualized using Circos v0.68 (36).

Functional annotations were assigned to the most significant putative ancestor of each microProtein candidate family using Protein Analysis through Evolutionary Relationships (PANTHER v13.1; www.pantherdb.org/). One-sided Fisher’s exact test for overrepresentation was performed with R version 3.4.4 against all annotated proteins of the *B. distachyon* v3.1 genome.

### Generation of Transgenic Plants.

The synthetic microProtein-OX plants were generated by amplification DNA encoding conserved domains of *Arabidopsis* orthologous protein using primers listed in the *SI Appendix*, Table S1. The PCR-amplified fragments were cloned into pDONR207 (Invitrogen) and recombined into pJAN33 binary vector, which was constructed from the pPAM (GenBank acc. no. AY027531), has a FLAG tag at N terminus for protein detection, and has Gateway recombination sites to facilitate the cloning of target gene fragments. These constructs driven by the double CAMV35S promoter, were used to transform Col-0 to generate overexpression lines using *Agrobacterium tumefaciens*-mediated transformation with floral dipping. The resulting transgenic T1 lines were screened by qPCR for synthetic *microProtein*-overexpressing lines. Homozygous *microProtein*-overexpressing T3 lines were used for phenotypic analyses.

To generate *LNJ-OX* plants, the *LNJ* coding sequence was amplified from *Brachypodium* immature spikelet complementary DNA (cDNA) using primers listed in the *SI Appendix*, Table S1 and cloned into pDONR207 (Invitrogen). The coding sequence was subsequently recombined into binary destination vector pIPKb003 (GenBank: EU161569) for overexpression. Transgenic Brachypodium plants were obtained by *Agrobacterium*-mediated transformation (37). Briefly, immature embryos were prepared and calli were induced on Linsmaier and Skoog (LS) basal medium supplemented with 2.5 mg/L 2,4-D. Calli were cocultivated with the *Agrobacterium* AGL1 strain containing the binary plasmid. Transgenic calli were selected on hygromycin medium. After shoot and root regeneration, the resulting plantlets were transferred to a regular greenhouse for continued growth and seed collection. Transgenic T2 lines were analyzed for growth phenotypes and *LNJ* expression levels. Increased branching correlated with elevated *LNJ* expression levels. Finally, homozygote T3 progeny was obtained that showed increased tilling.

The *BdLNJ-OX* plasmid was also transformed into barley (*H. vulgare* cv. Golden Promise) and we generated >15 independent transgenic lines (T0) by *A. tumefaciens-*mediated embryo transformation (38). Transgenic plants were tested by analyzing the levels of *LNJ* expression by qPCR. Transgenic T2 plants with high *LNJ* levels were subsequently used for the scoring of agronomic traits.

Finally, we also transformed the *BdLNJ-OX* construct in rice (Nipponbare) and obtained >20 independent transgenic lines. For rice transformation, we induced immature embryonic calli on CHU (N6) medium supplemented with vitamins and 2 mg/L 2,4-D. After cocultivation with the *Agrobacterium* AGL1 strain containing the binary plasmid, the transgenic calli were selected on hygromycin medium. Transgenic plantlets were obtained by inducing shoots and roots and subsequently used for trait scoring. Branching was analyzed as described before (39).

The *afp2*-CRISPR deletion mutants were generated using the pKI1.1R vector system (40). Specific single guide RNAs were designed using CRISPR-P (41). To achieve mutants with larger deletions in the coding region, individual pKI1.1R vectors containing different gene-specific sgRNAs were pooled and transformed into wild-type Col-0. The red fluorescence seed coat marker was used for selection of transgenic seeds and PCR was used to detect larger deletions.

### Yeast-Two and -Three Hybrid Experiments.

For yeast-two-hybrid analysis, fusions of the Gal4-binding domain (BD) to respective proteins were achieved by cloning the respective coding sequence into the pGBKT7-GW vector. BD-fusion constructs were transformed into the pJ69-4α yeast strain. Fusions to the Gal4-activation domain (AD) were achieved by cloning respective coding sequences into the pGADT7-GW vector. AD-fusions were transformed into the YM4271 MATa strain. The presence of the plasmids in the strains was verified by PCR, and positive strains were mated for 2 d at 28 °C and then selected on dropout media lacking tryptophan and leucine. Positive colonies were screened on selective triple dropout medium (-Trp, -Leu, -His). For the yeast-three-hybrid assay, the *BdNINJA* coding sequence was recombined into a pGBKT7-GW vector and *BdLNJ* into the pDRf1-GW yeast expression vector. The empty vectors or the vectors containing the respective coding sequences were cotransformed into the pJ69-4α yeast strain, and positive colonies were selected on dropout media lacking Trp and Ura. The *pGADT7-BdNINJA* vector was transformed into a YM4271 MATa strain and selected on dropout media without Leu. The presence of the plasmids in the strains was verified by PCR and positive strains for each transformation were mated for 2 d at 28 °C and then selected on dropout media without Trp, Leu, and Ura. Positive colonies were screened on selective media without Trp, Leu, Ura, and His with additional 2.5 mM 3-aminotriazole.

### Analysis of Gene Expression.

Total RNAs were isolated from leaves of *Arabidopsis*, *Brachypodium*, barley, and rice plants using a Spectrum Plant Total RNA Kit according to the manufacturer’s instructions (Sigma). For *LNJ* and synthetic *microProtein* expression, rosette leaves of 4-wk-old plants grown at 22 °C were collected for RNA preparation. For *Brachypodium*, barley, and rice *LNJ* gene expression, RNA was prepared from leaves of 4-wk-old plants grown in each appropriate growth condition.

Total RNAs were also prepared from leaves of 4-wk-old Brachypodium plants treated by methyl jasmonate for analysis of gene expression in response to JA. For the MeJA application, we followed a treatment regime published earlier (42). In brief, three independent plant lines with 12 individuals each were treated after 4 wk by spraying with methyl jasmonate (100 μM in 0.15% Tween) or a mock solution (0.15% Tween). Treatments were performed over an interval of 3 d over a period of 2 wk. After the fifth application, plants were harvested for RNA analysis 30 min after treatment and growth and development changes were recorded. Gene expression analysis was carried out by qRT-PCR for which we converted 1 μg of total RNA into cDNA using the RevertAid First Strand cDNA Synthesis Kit (Thermo Fisher Scientific). All qRT-PCR reactions were performed in triplicates on a CFX384 Real-Time System Cycler (Bio-Rad) with KAPA SYBR FAST qPCR Master Mix Bio-Rad iCycler (KAPA Biosystem) according to the manufacturer’s instructions. We used a two-step amplification procedure (50 cycles à 95 °C for 5 s and 60 °C for 30 s). Relative expression levels were calculated using the ΔΔCT method. Oligonucleotide sequences are listed in the *SI Appendix*.

### RNA-Seq and Bioinformatics Analysis.

The 4-wk-old *Brachypodium* leaves from the wild-type, *BdLNJ-OX* plants (*n* = 12 plants for each sample) were collected to extract the total RNA. For *Arabidopsis*, leaves of 20-d old soil-grown *Arabidopsis* plants were collected for RNA extraction. In total, we collected two biological replicates for each genotype and treatment. The mRNA library preparation and sequencing was performed at Novogene Company Limited (Hong Kong).

Sequencing libraries were prepared using the Illumina TruSeq RNA Library Preparation Kit according to the manufacturer’s recommendations. Libraries were sequenced on the Illumina HiSeq2000 platform, and between 17 and 21 million read pairs per sample were obtained. Approximately 377 million paired-end reads were loaded into Galaxy version 15.05.rc1 (43–45), and quality was assessed using FastQC (version 0.10.1). Tophat2 (version 2.0.9) aligned above 85% of read pairs of each sample correctly to Brachypdium genome. Galaxy’s Cufflinks package (version 0.0.7) was employed for differentially expressed gene calling (cutoff q-value of 0.05) (46). Gene expression levels were calculated as FPKM (Fragments Per Kilobase Million), and differentially expressed genes were chosen based on a false discovery rate (FDR) ≤ 0.001 and ≥2 fold change. A heat map of differentially expressed genes on FPKM values was visualized by the online tool Heatmapper (www.heatmapper.ca) using average linkage clustering and Euclidean distance measurement. All data, including the processed data files, have been submitted to the Gene Expression Omnibus (https://www.ncbi.nlm.nih.gov/geo/): accession number GSE124493.

### MeJA Treatment.

For mMeJA treatment, wild-type *Brachypodium* Bd21-3 and *BdLNJ*-OX plants were grown in the growth chamber with a growth condition described as above. For the control group, plants were sprayed with 50 mL of 0.15% Tween-20 solution (labeled as “Mock”). For the MeJA treatment group, plants were sprayed with 50 mL of a 100 μM MeJA solution (95% MeJA dissolved in 0.15% Tween-20 solution; labeled as “JA”). About 8 plants each line, 4-wk-old-plants were treated every 2 d for a week (3 times).

### Phytohormone Analysis by UHPLC/TQ-MS.

Phytohormones were extracted from around 200 mg (fresh weight, FW) plant material ground in liquid nitrogen with 1.25 mL 80% (vol/vol) methanol containing 4 μL of internal standard mix containing 88 pmol (2H5)tZ and 216 pmol (2H6)ABA (abscisic acid) (both purchased from OlChemIm). Samples were thoroughly vortexed, incubated for 30 min at 4 °C, and centrifuged (20.000 g, 4 °C, 15 min). Supernatants were passed through C18 columns (Phenomenex, Strata C18-E, 8B-S001-FBJ) after pre-equilibration with three times 3 mL 80% (vol/vol) methanol, and flow-throughs were collected and kept on ice. Extraction was repeated with 1.25 mL 80% (vol/vol) methanol and second extracts were passed through the same columns. The combined extracts were concentrated using a SpeedVac. The residues were dissolved in 1 mL 20% (vol/vol) methanol by brief sonication and filtered (MultiScreenHTS; EMD Millipore, cat no. MSGVN 2250). Phytohormones were analyzed by ultra-performance liquid chromatography-triple quadruple mass spectrometer (UHPLC/TQ-MS) on an AdvanceTM-UHPLC/EVOQTMElite-TQ-MS instrument (Bruker) equipped with a C-18 reversed phase column (Kinetex 1.7 u XB-C18, 10 cm × 2.1 mm, 1.7 µm particle size, Phenomenex) by using a 0.05% formic acid in water (vol/vol), pH 4.0 (solvent A)–methanol (solvent B) gradient at a flow rate of 0.4 mL/min at 40 °C. The gradient applied was as follows: 10 to 50% B (15 min), 50% (2 min), 50 to 100% B (0.1 min), 100% B (2.9 min), 100 to 10% B (0.1 min), and 10% B (5 min). Compounds were ionized by electrospray ionization with a spray voltage of +4,500 V and −4,000 V in positive and negative mode, respectively; heated probe temperature was 350 °C, cone temperature was 300 °C. Quantification was based on response factors relative to (2H5)tZ (positive mode) and (2H6)ABA negative mode. The individual hormones were monitored based on the following multiple reaction monitoring (MRM) transitions: (2H5)tZ, (+) 225 > 137 [15 V]; (2H6)ABA, () 269 > 159 [7 V]; ABA, (−) 263 > 153 [7 V]; 1-aminocyclopropane-1-carboxylic acid, (+) 102 > 56 [15 V]; indole-3-acetic acid, (+) 176 > 130 [10 V]; indole-3-aldehyde, (+) 146 > 118 [15 V]; isopentenyladenine-riboside, (+) 336 > 2014 [15 V]; JA-Ile, (−) 322 > 130 [17 V]; SA, (−) 137 > 93 [20 V]; trans-zeatin, (+) 220 > 136 [15 V]; tZ7G/tZ9G/tZOG, (+) 382 > 220 [17 V]; tZR, (+) 352 > 220 [15 V]; tZROG, (+) 514 > 382 [15 V]. tZ7G, tZ9G, and tZOG were distinguished based on retention times in comparison to those of known standards.

## Supplementary Material

Supplementary File

Supplementary File

Supplementary File

## Data Availability

RNA-seq data have been deposited in Gene Expression Omnibus (accession no. GSE124493 and GSE155487).
