# Supplementary material for: Heterologous microProtein expression identifies LITTLE NINJA, a dominant regulator of jasmonic acid signaling
Source: Proc Natl Acad Sci U S A. 2020 Oct 8;117(42):26197–205. doi: 10.1073/pnas.2005198117 (PMC7584889; doi:10.1073/pnas.2005198117)
Supplement: Supplementary File [file pnas.2005198117.sapp.pdf]

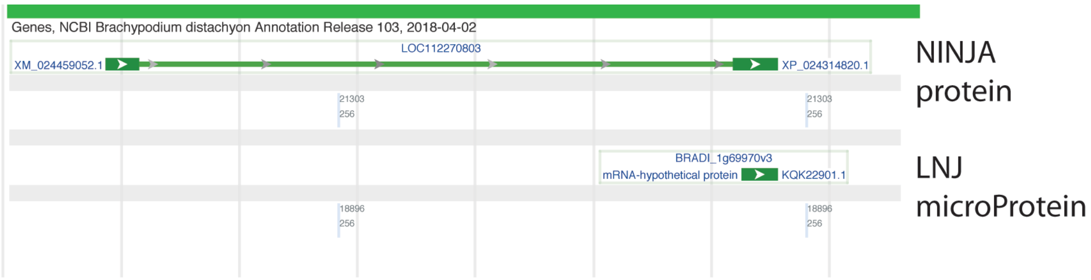

Supplementary Figure S1. Genome browser snapshot showing the Brachypodium LOC112270803 gene. Annotated in the Brachypodium genome was BRADI1g69970v3 encoding the LNJ microProtein. In-depth analysis revealed that LNJ is a splice variant of a larger gene encoding a NINJA-like protein.

www.pnas.org/cgi/doi/10.1073/pnas.2005198117

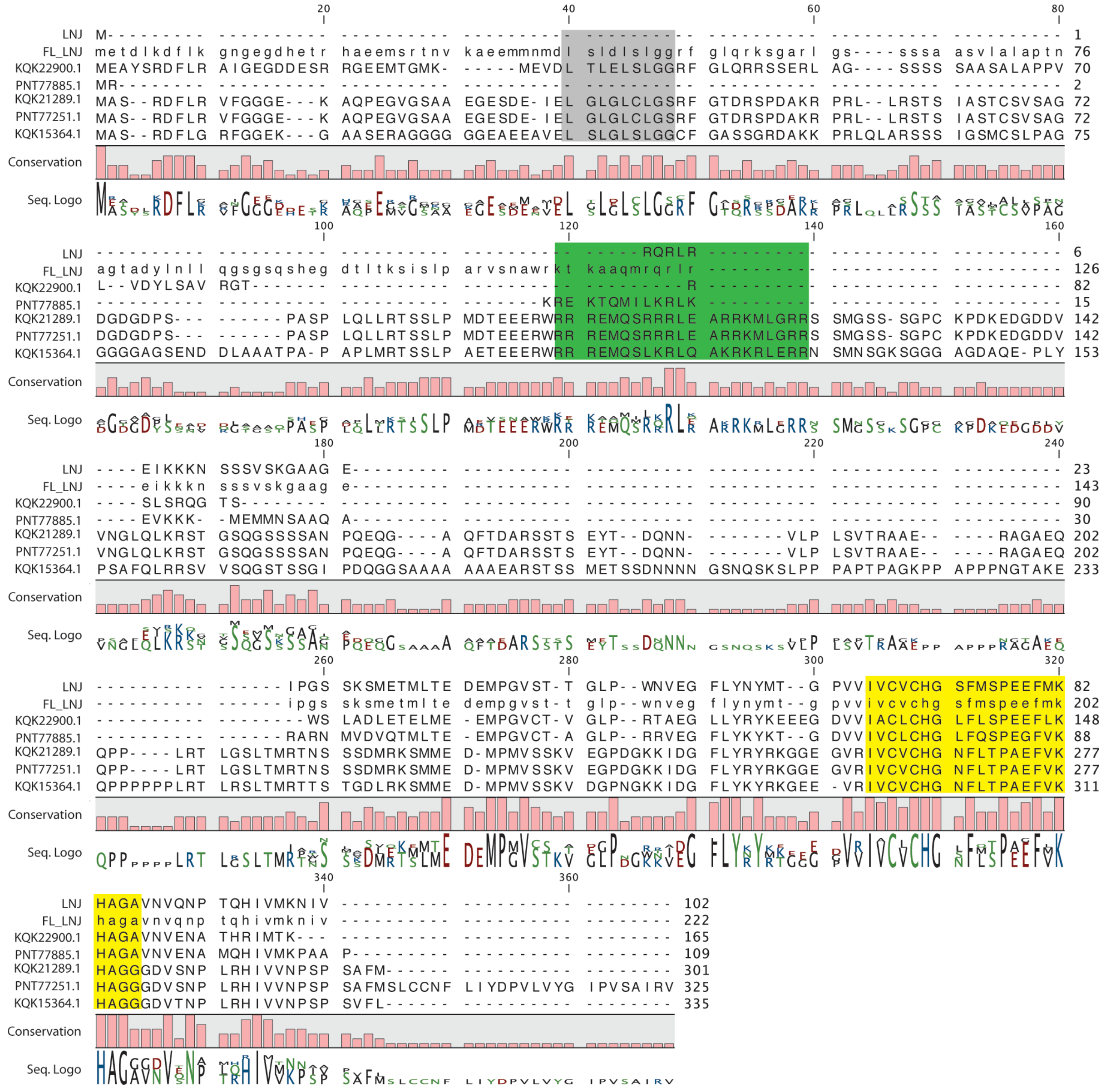

Supplementary Figure S2. LNJ and Brachypodium NINJA-like proteins multiple sequence alignment. Multiple protein sequence alignment of LNJ with related proteins was carried out using CLC Workbench 7 align tools. Gray box indicates Ethylene-responsive element binding factor-associated Amphiphilic Repression (EAR) motif, defined by the consensus sequence patterns of either LxLxL or DLN xxP. The green box highlights domain B and the yellow box highlight domain C.

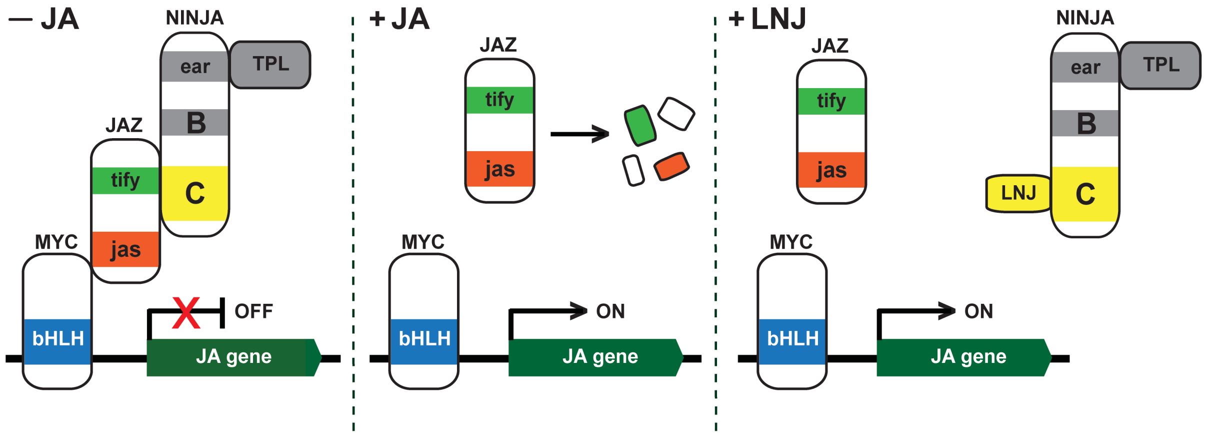

Supplementary Figure S3. Hypothetical model for the action mechanism of LNJ microProtein in the JA signaling. In the absence of jasmonic acid (left), JAZ proteins interacting through the TIFY motif with domain C of NINJA to keep MYC factors in a repressed state. The EAR motif of NINJA is essential for the interaction with the TPL co-repressors. In the presence of jasmonic acid (middle), JAZ proteins subsequently release NINJA/TPL complex from the MYC factors and induce jasmonate-responsive genes which mediate various responses including pathogen defense. In the case of introducing LNJ in the JA signaling (right), NINJA protein can interact with LNJ microProtein via its domain C. NINJA function can thereby be modulated by LNJ microProtein in various JA mediated response.

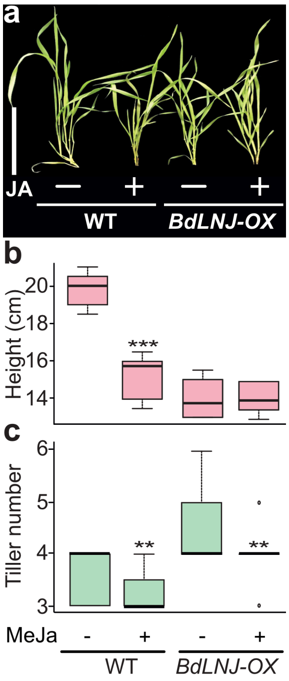

Supplementary Figure S4. MeJA treatment of wild type and BdLNJ-OX plants. **a** Pictures of wild-type Bd21-3 and the strongest overexpressing BdLNJ-OX line. Scale bar, 10 cm. **b**, **c** Quantification of height (**b**) and tiller number (**c**) depicted as box plots in wild-type and three independent wild-type Bd21-3, the strongest overexpressing BdLNJ-OX line +/- MeJA treatment. *P<0.05, **P<0.005, ***P<0.0005 determined by Student's T-Test. N = 15-20.

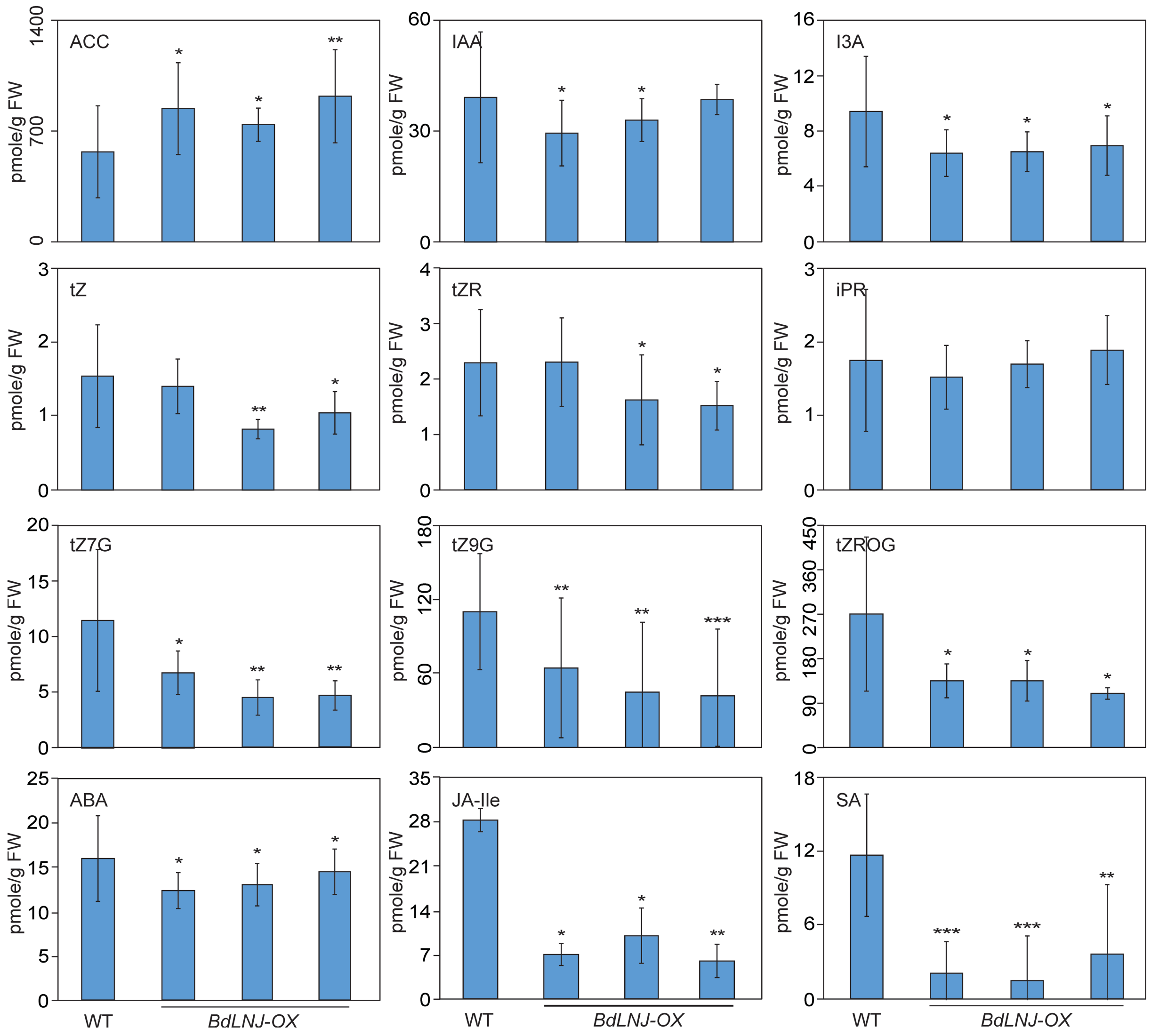

Supplementary Figure S5. Endogenous hormone levels in Brachypodium wild-type and BdLNJ-OX plants. The hormone levels in leaves were measured using UHPLC/TQ-MS. ACC, 1-aminocyclopropane-1-carboxylic acid; IAA, Indole-3-acetic acid; I3A, Indole-3-carboxaldehyde; tZ, trans-zeatin; tZR, tZ riboside; iPR, N6-(Δ2-isopentenyl) adenine (iP), iP riboside; tZ7G, Trans-zeatin-7-glucoside; tZ9G, Trans-zeatin-9-glucoside; tZROG, tZR-O-glucoside; ABA, abscisic acid; JA-Ile, jasmonoyl-L-isoleucine; SA, salicylic acid. Values are the means ±SD. N = 6-8. *P<0.05, **P<0.005, ***P<0.0005, determined by Student's T-Test.

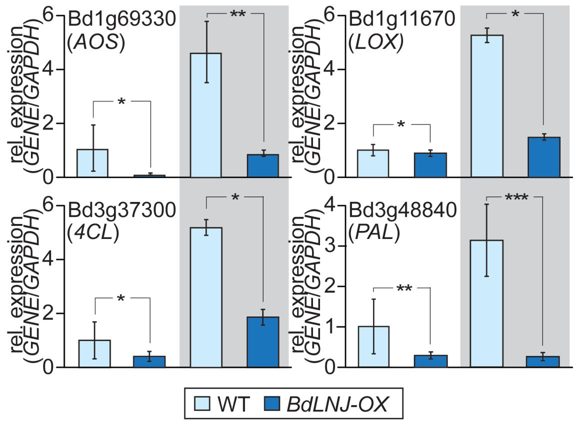

Supplementary Figure S6. Relative expression levels of JA biosynthesis and response genes in wild-type and BdLNJ-OX plants in MeJA treatment. Relative expression levels of JA biosynthesis/response genes in wild-type and the strongest overexpressing BdLNJ-OX line determined by qRT-PCR with MeJA treatment (gray boxed). PAL is an ethylen response gene that is also induced by methyl-jasmonate (see Kouzai et al., BMC Plant Biology, 2016). Expression levels, determined as relative quantities and normalized against Brachypodium GAPDH gene, are depicted as bar graphs. *P<0.05, **P<0.005, ***P<0.0005 determined by Student’s T-Test.

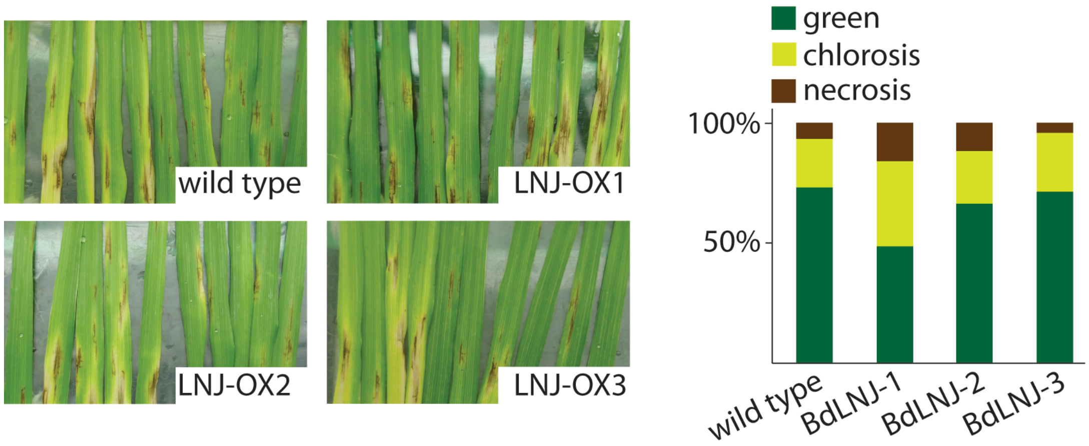

Supplementary Figure S7. Determination of pathogen susceptibility of wild type and transgenic barley plants. Wild type and transgenic *LNJ-OX* plants were infected with *Pyrenophora teres* (CP2189). Chlorotic and necrotic leaf areas were measured and percentages were calculated using Assess 2.0 from APS.

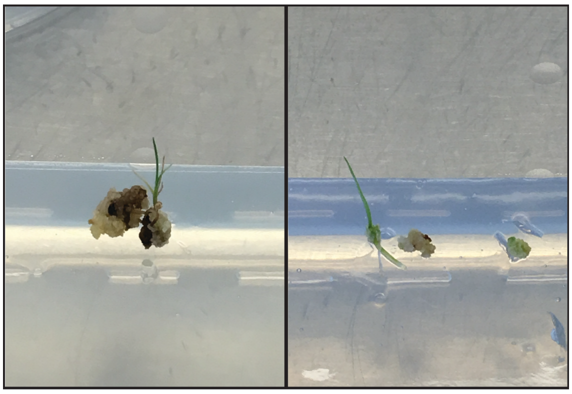

Supplementary Figure S8. Regeneration of transgenic plants transformed with a CRISPR/Cas9 guide RNAs targeting the *LNJ* gene. After isolation of transgenic calli, shoots were regenerated but transgenic plants failed root regeneration and died soon after. This indicated that the LNJ gene is essential for plant development.

Supplemental Table 1: Oligonucleotides used in this study.

Primer	Gene ID	Sequence	Purpose
BdLNJ F<br>BdLNJ R	BD1G69970	ggggacaagtttgtacaaaaaagcaggcttcATGCGGCAGAGGCTCAGGGA<br>ggggaccactttgtacaagaaagctgggtcCTAGACTATGTTTTTC	Cloning CDS
LNJ F<br>LNJ R	AT1G13740	ggggacaagtttgtacaaaaaagcaggcttcATGGCTACGTCTACTGGTTTGG<br>ggggaccactttgtacaagaaagctgggtcTTAAAAGGTCGAAGAAGAGGTG	Cloning CDS
BTB-POZ F<br>BTB-POZ R	AT5G19000	ggggacaagtttgtacaaaaaagcaggcttcATGCTCCATTTTATCTACTGGGACG<br>ggggaccactttgtacaagaaagctgggtcTCACCTCGCCACATACTGCAATAGCTCC	Cloning CDS
BTB-ZF F<br>BTB-ZF R	AT3G42170	ggggacaagtttgtacaaaaaagcaggcttcATGGCTAGAGACATCC<br>ggggaccactttgtacaagaaagctgggtcCTATGCTTCAGATTTGATGG	Cloning CDS
SERPIN F<br>SERPIN R	AT2G26390	ggggacaagtttgtacaaaaaagcaggcttcATGGTTGATTCGCCTTCGAACGG<br>ggggaccactttgtacaagaaagctgggtcTCAATGTTTAGAAGGATCAAGAACTTGACC	Cloning CDS
XHXS F<br>XHXS R	AT3G12550	ggggacaagtttgtacaaaaaagcaggcttcATGTGGGAGGAATATCTTAAGGACCC<br>ggggaccactttgtacaagaaagctgggtcTTATGACTTGAGATGCTTCGC	Cloning CDS
BdJAZ1 F<br>BdJAZ1R	BRADI_1g21490	ggggacaagtttgtacaaaaaagcaggctgcATGGCGGCTTCCACGAGGCC<br>ggggaccactttgtacaagaaagctgggtcCTAAAGCTCAGGTTTGGCGG	Cloning CDS
BdNINJA F<br>BdNINJA R	BRADI_2g11790	caccATGGAGGATGGCCTTGAGCTTAG<br>CTAGTTTTGGGCTGAGGCTGC	Cloning CDS
BdLNJ qF<br>BdLNJ qR	BD1G69970	ACCAGGGAGTAGCAAGAGCA<br>CAGCTCCAGCATGCTTCATA	qRT-PCR
LNJ qF<br>LNJ qR	AT1G13740	GGTTGATATGCCTTGTGTGTTTACT<br>AAGAAGAGGTGTTGACAACGATATG	qRT-PCR
BTB-POZ qF<br>BTB-POZ qR	AT5G19000	GAGCGGCTTAAAGCAATCTG<br>CTGCAAACAATGATGCTGCT	qRT-PCR
BTB-ZF qF<br>BTB-ZF qR	AT3G42170	TCAGCTGCAGCCTTTGACTA<br>CTCAAGAAGCCATTCCCTTG	qRT-PCR
SERPIN qF<br>SERPIN qR	AT2G26390	GCCTTCGAACGGTGATAAAC<br>ATCAAACATTGGGGCATCAT	qRT-PCR
XHXS qF<br>XHXS qR	AT3G12550	CCCAGATTGGCATCCTTTTA<br>TCCCCCAGTTCATTCTTCAG	qRT-PCR
AtGAPDH qF<br>AtGAPDH qR	AT1G13440	AAAGTGTTGCCATCCCTCAA<br>TCGGTAGACACAACATCATCCT	qRT-PCR
PR14 qF<br>PR14 qR	BD1G19470	CCATCTGCGACATCTCCAGC<br>CGCTCTTGTACCGGCAGAG	qRT-PCR
PR2 qF<br>PR2 qR	BD3G07960	GTTCAACGTCGCCATGGAC<br>TGGGTCTTAACGTCGTTGGG	qRT-PCR
DOF qF<br>DOF qR	BD3G51510	GAATAATCAACCACCGGCGC<br>GCAGTTGATCGCCTTCTCCT	qRT-PCR
MYB86 qF<br>MYB86 qR	BD2G17982	TGGAACAGCTGCCTCAAGAA<br>CGGCTTCTGGTCTTCGTCTT	qRT-PCR

JMT qF<br>JMT qR	BD2G47550	TCTTCCCCTTGTTGATCGGC<br>GTTCAACGTCGCCATGGAC	qRT-PCR
SnR-Kinase qF<br>SnR-Kinase qR	BD4G30390	GGCATGTCGGAGCAGATGAA<br>CTCCAGGGCCAGGTAGATCT	qRT-PCR

Primer	Gene ID	Sequence	Purpose
MADS qF<br>MADS qR	BD1G21980	CCAGAATCTCAGCCGTCTCC<br>CTGATGGCACCGATATCCCC	qRT-PCR
AGL7 qF<br>AGL7 qR	BD1G08340	AAGAAGGCGCACGAGATCTC<br>CATACATGAGTCGGTGGCGA	qRT-PCR
AOS7 qF<br>AOS7 qR	BD1G69330	ACCGCCTGGACTTCTACTAC<br>GAGGTTCTTCTTCTCCACCT	qRT-PCR
LOX qF<br>LOX qR	BD1G11670	TCAACTTGCCCTTTCCACATG<br>GCAAACCGGATTAACTCCTGC	qRT-PCR
4CL qF<br>4CL qR	BD3G37300	GTTCGAGACGGTGAGGATGTTC<br>CATACTTTCCAACAGCGTCGC	qRT-PCR
PAL qF<br>PAL qR	BD3G48840	TCTTTGAGGCAAACATTCTT<br>ATAGCAGCAGCCTCTATTTG	qRT-PCR
BdGAPDH qF<br>BdGAPDH qR	BD3G14040	TTGCTCTCCAGAGCGATGAC<br>CTCCACGACATAATCGGCAC	qRT-PCR
HvGAPDH qF<br>HvGAPDH qR	HV1G074690	GCCAAGACCCAGTAGAGC<br>CACATTTATTCCCATAGACAAAGG	qRT-PCR
OsACTIN qF<br>OsACTIN qR	OS08T0126300	CCCAGGAATTGCTGACCGTA<br>TTTTCTCTCTGGCGGTGCA	qRT-PCR

Supplementary data sets

Supplementary dataset 1. Identification of differentially expressed genes using RNA-seq. The file contains two spreadsheets containing differentially expressed genes in Col-0 wilt type versus transgenic *LNJ-OX* plants and differentially expressed genes in Col-0 wilt type versus *jazD* mutant plants.

Supplementary dataset 2. Identification of differentially expressed genes in Brachypodium. Shown is own spreadsheet containing all comparision of the four different datasets (wild type mock; wild type MeJA-treament; *LNJ-OX* mock; *LNJ-OX* MeJa-treatment).
